# Lumen-apposing metal stent for peripancreatic fluid collection contributes to early improvement of nutritional status: A multicenter retrospective study (YCR-P001)

**DOI:** 10.1097/MD.0000000000048020

**Published:** 2026-03-13

**Authors:** Takanori Tsuyama, Shigeyuki Suenaga, Harumi Suehiro, Ukyo Shinagawa, Kaori Hamamoto, Shoko Tabara, Shuhei Shinoda, Shogo Amano, Manabu Sen-Yo, Noriko Ishigaki, Michitaka Kawano, Hirofumi Harima, Taro Takami

**Affiliations:** a Department of Gastroenterology and Hepatology, Yamaguchi University Graduate School of Medicine, Ube, Yamaguchi, Japan; b Department of Gastroenterology, Tokuyama Central Hospital, Shunan, Yamaguchi, Japan; c Department of Gastroenterology, Saiseikai Yamaguchi General Hospital, Yamaguchi, Yamaguchi, Japan; d Department of Gastroenterology, Saiseikai Shimonoseki General Hospital, Shimonoseki, Yamaguchi, Japan.

**Keywords:** controlling nutritional status, endoscopic ultrasound-guided transluminal drainage, lumen-apposing metal stent, peripancreatic fluid collection, plastic stent, prognostic nutritional index

## Abstract

Acute pancreatitis causes malnutrition due to severe systemic inflammatory response and organ failure and is associated with increased susceptibility to severe infections and complications such as peripancreatic fluid collection (PFC). PFC is treated using endoscopic ultrasound-guided transluminal drainage (EUS-TD). Lumen-apposing metal stents (LAMSs) have high drainage efficiency and are useful; however, their impact on improving nutritional status and long-term prognosis is unclear. We aimed to evaluate the clinical outcomes and nutritional status after LAMS versus plastic stent (PS) in patients with PFC. This multicenter retrospective study included 59 patients who underwent EUS-TD (18 LAMS and 41 PS) for PFC between December 2018 and March 2023. Nutritional indices (prognostic nutritional index [PNI] and modified controlling nutritional status [M-CONUT]) were evaluated using blood tests 2, 4, and 8 weeks postoperatively, and compared using analysis of covariance, with pretreatment values as covariates. Endoscopic procedure time (21.0 vs 48.0 min; *P* <.001) and time to diet initiation (3.0 vs 5.0 days; *P* = .04) were significantly shorter in the LAMS group. There were no differences in the clinical success rate (88.9 vs 85.4%; *P* >.99), number of procedures (2.0 vs 2.0; *P* = .68), time to discharge after the procedure (28.5 vs 36.0 days; *P* = .19), adverse events (16.7 vs 14.6%; *P* >.99), or recurrence rate (16.7 vs 17.1%; *P* >.99). Post-procedure PNI (2 weeks: 4.5 vs −0.1; *P* = .09, 4 weeks: 7.2 vs 0.7; *P* = .03, 8 weeks: 11.0 vs 5.1; *P* = .05) and M-CONUT (2 weeks: −1.7 vs 0.5; *P* = .03, 4 weeks: −2.2 vs 0.4; *P* = .03, 8 weeks: −3.5 vs −1.1; *P* = .03) showed better improvement in the LAMS group. LAMS group show earlier improvement of nutrition status than PS group in this study.

## 1. Introduction

Acute pancreatitis has a poor prognosis, with a fatality rate of 30% in severe cases with multiple organ failure and pancreatic necrosis.^[1]^ With the establishment of initial treatments in recent years, the mortality rate in the early phase of the disease has declined. However, the mortality rate in the late phase of the disease remains the same; therefore, appropriate management of late complications is important.^[2]^ Acute pancreatitis causes a severe systemic inflammatory response, necrosis, and organ failure, resulting in increased energy requirements and protein catabolism. In addition, prolonged fasting exacerbates malnutrition and negative nitrogen balance and is associated with increased mortality from infectious complications and multi-organ failure.^[3,4]^ Therefore, early intervention with nutritional therapy is important.^[5]^ Enteral nutrition contributes to the maintenance of intestinal mucosal barrier function compared with full intravenous nutrition. Several randomized controlled trials and meta-analyses have reported significant reductions in mortality, infectious complications, multiple organ failure, and surgical interventions.^[6]^

Peripancreatic fluid collection (PFC) is a frequent late complication of pancreatitis, and appropriate nutritional therapy is important in its initial treatment.^[7]^ Malnutrition is closely related to increased susceptibility to infection, severity of infection, and complications due to decreased immunocompetence. PFC requires interventional treatment in cases of infectious complications, biliary tract or gastrointestinal tract stenosis, or a tendency to enlarge.^[8]^ Recently, a step-up approach starting with less-invasive procedures has been recommended because of its lower complication and mortality rates.^[9]^ Endoscopic ultrasound-guided transluminal drainage (EUS-TD) was first described by Grimm et al in 1992.^[10]^ Initially, plastic stents (PSs) and fully covered self-expandable metallic stents (FCSEMSs) were initially used in EUS-TD. However, a PS has the disadvantage of a small inner diameter, which can lead to drainage failure due to stent occlusion. FCSEMSs also have problems with bleeding due to stent migration and stent edge contact.^[11]^ The development of the lumen-apposing metal stent (LAMS), a device specifically designed for EUS-TD, has significantly changed the treatment strategy. LAMS are designed with bilateral wide flanges that provide stable lumen-to-lumen apposition and reduce migration, in contrast to conventional tubular FCSEMSs. LAMS also have a larger diameter (typically 10–20 mm), facilitating endoscopic necrosectomy and efficient drainage, and are deployed using a dedicated delivery system under EUS guidance.^[11,12]^ The advantages of LAMS over conventional PS and FCSEMS are its large diameter, superior drainage efficiency, simplicity of the procedure, and transition to endoscopic necrosectomy. In 2012, Itoi et al reported the results of a clinical trial of LAMS for pancreatic pseudocysts (PPCs) in which all patients were successfully treated without serious complications.^[11]^ Subsequently, several studies comparing LAMS with PS and FCSEMS have reported that LAMS is useful owing to its higher clinical success rates and shorter procedure and hospitalization times.^[13–16]^ However, how the choice between LAMS and PS affects nutritional status improvement and long-term prognosis has not been fully analyzed.

We hypothesized that LAMS may improve long-term prognosis by rapidly improving inflammation in PFC, allowing for earlier oral intake, thus improving nutritional status and preventing re-infection in PFC. This study aimed to examine the nutritional status, clinical outcomes, and adverse events after the procedure with LAMS versus PS in patients who underwent EUS-TD for PFC at a multicenter setting.

## 2. Methods

### 2.1. Study design and patient selection

This study was conducted in accordance with the Declaration of Helsinki and approved by the Institutional Ethics Committee of the Yamaguchi University (Approval number: H2023-208). This study adhered to the STROBE guidelines for observational studies. Patients who underwent EUS-TD for PFC at Yamaguchi University Hospital and 3 affiliated hospitals between December 2018 and March 2023 were included in this retrospective study. As this was a retrospective observational study, no formal sample size calculation was performed. The sample size was determined by the number of eligible patients during the study period, and the study was exploratory in nature. PFC was classified according to the 2012 revised Atlanta Classification.^[17]^ EUS-TD was indicated for patients with signs of infection, jaundice, gastrointestinal obstruction, or other symptoms; those who have PFC with a tendency to increase over time; or those with no tendency to regress. Patients who underwent percutaneous drainage or surgical necrosectomy prior to EUS-TD, those with a follow-up of <1 month, or those with a severe bleeding tendency were excluded from this study. Since 2017, when LAMS became available in Japan, patients with PFC size ≥ 60 mm, distance from the stomach wall <10 mm, and liquid component inside the cyst ≥ 70% were indicated for LAMS. PS was selected for patients who did not meet the LAMS criteria. The patients were divided into 2 groups, the LAMS and PS groups, and compared retrospectively.

Patient characteristics, such as age; sex; drinking history; smoking history; cause, classification, and maximum diameter of PFC; presence of infection; prognostic nutritional index (PNI); and modified controlling nutritional status (M-CONUT) score, were extracted from the medical records. PNI was calculated using the following formula proposed by Onodera et al: 10 × serum albumin (g/dL) + 0.005 × total lymphocyte count (TLC) (per mm^3^).^[18]^ The controlling nutritional status (CONUT) score is calculated by summing the scores of serum albumin, TLC, and total cholesterol as follows: serum albumin (≥3.5 g/dL [0 point]; 3.0–3.4 g/dL [2 points]; 2.5–2.9 g/dL [4 points]; or <2.5 g/dL [6 points]), TLC (≥1600 cells/mm^3^ [0 point]; 1200–1599 cells/mm^3^ [1 point]; 800–1199 cells/mm^3^ [2 points]; or <800 cells/mm^3^ [3 points]), and total cholesterol level (≥180 mg/dL [0 point], 140–179 mg/dL [1 point], 100–139 mg/dL [2 points], or <100 mg/dL [3 points]).^[19]^ In Japan, the M-CONUT score, in which total cholesterol is replaced by hemoglobin concentration (male ≥ 13.0 g/dL, female ≥ 12.0 g/dL [0 point]; male 10.0–12.9 g/dL, female 10.0–11.9 g/dL [1 point]; 8.0–9.9 g/dL [2 points]; or <8.0 g/dL [3 points]) is widely used.^[20]^

Technical and clinical success rates, endoscopic procedure time at initial treatment, time from initial treatment to resumption of oral intake, number of procedures, time from initial treatment to discharge, adverse events, mortality rates, recurrence rates, and nutritional status were examined. Technical success was defined as the successful placement of LAMS or PS in the PFC.^[21]^ Clinical success was defined as improvement in clinical symptoms and at least a 50% reduction in PFC within 60 days after the initial drainage.^[22]^ The number of procedures was defined as the number of procedures required to improve the PFC, including stent addition or replacement, endoscopic necrosectomy, percutaneous drainage, and surgical necrosectomy. Oral intake was initiated after the procedure, with a trend toward improvement in clinical symptoms and inflammatory findings. Recurrence was defined as the worsening of clinical symptoms or an increase in PFC after discharge from the hospital that required therapeutic intervention. Abdominal circumference, visceral fat area, subcutaneous fat area, and L3 skeletal muscle index (L3SMI) were measured in cases where computed tomography (CT) imaging was available 4 weeks after the procedure; L3SMI was calculated as the skeletal muscle area at the L3 level (cm^2^)/height squared (m^2^).^[23]^ The PNI and M-CONUT score were measured 2, 4, and 8 weeks after the procedure, and the amount of change from before the procedure was calculated. Patients with anemia due to hemorrhage or blood transfusions were excluded from nutritional evaluation.

### 2.2. Procedure

Convex ultrasound endoscopes (UCT240 and UCT260; Olympus Medical Systems, Tokyo, Japan) were used for EUS-TD.

In the LAMS group, the Hot AXIOSTM system (Boston Scientific, Marlborough) was used as the device in all patients. The PFC was drawn from the stomach or duodenum, and the LAMS was placed at a puncture site selected where the distance from the gastrointestinal wall to the cyst wall was within 10 mm, and the diameter of the cyst on the puncture line was at least 40 mm. A 7 Fr, pigtail PS (ERBD set®, Hanaco Medical, Saitama, Japan; Through & Pass®, Gadelius Medical, Tokyo, Japan) was placed in the PFC via the LAMS at the discretion of the treating physician.

In the PS group, the PFC was punctured with a 19 G puncture needle (EZ Shot 3 Plus®, Olympus Medical Systems; Expect®, Boston Scientific; EchoTip®, Cook Medical, Bloomington) and a 0.025 inch guidewire (EndoSelector®, Boston Scientific; Pathcourse®, Boston Scientific; VisiGlide2®, Olympus Medical Systems) were placed. A dilator (Soehendra® Biliary Dilation Catheter 6-10 Fr, Cook Medical; Cysto-Gastro-Set® 6 Fr, Endo-Flex Gmbh, Voerde, Germany) was used to dilate the fistula. A 7 Fr pigtail PS stent and a 6 Fr endoscopic nasobiliary drainage tube (ENBD) (FleximaTM ENBD Catheter, Boston Scientific) were used. The number of stents used and whether an external fistula was used were determined at the discretion of the treating physician.

### 2.3. Follow-up

CT imaging was performed 7 days after the initial EUS-TD to evaluate the effect of the treatment. Additional procedures were performed if the clinical symptoms improved or if the PFC did not shrink. The procedures were selected according to the pathology of the individual cases as follows: stent replacement/addition, endoscopic necrosectomy, percutaneous drainage, and surgical necrosectomy. If clinical symptoms and PFC improved in the LAMS group, the LAMS was removed 3 to 4 weeks after implantation, and replacement with a double-pigtail-type PS was attempted. In the PS group, the double-pigtail-type PS in implantation was retained indefinitely. Nutritional indices (PNI and M-CONUT) were evaluated using blood tests 2, 4, and 8 weeks postoperatively.

### 2.4. Statistical analysis

Continuous variables were summarized as mean ± standard deviation or median (range), and categorical variables as numbers and percentages. All statistical analyses were performed using JMP Pro version 16 (SAS Institute, Cary), and *P* <.05 was considered significant. Comparisons between the 2 groups were made using the Student *t*-, Mann–Whitney *U*, or Fisher exact probability test. Changes in nutritional indices, abdominal circumference, visceral fat area, subcutaneous fat area, and L3SMI were compared using analysis of covariance, with pretreatment values as covariates.

## 3. Results

**Figure 1.**
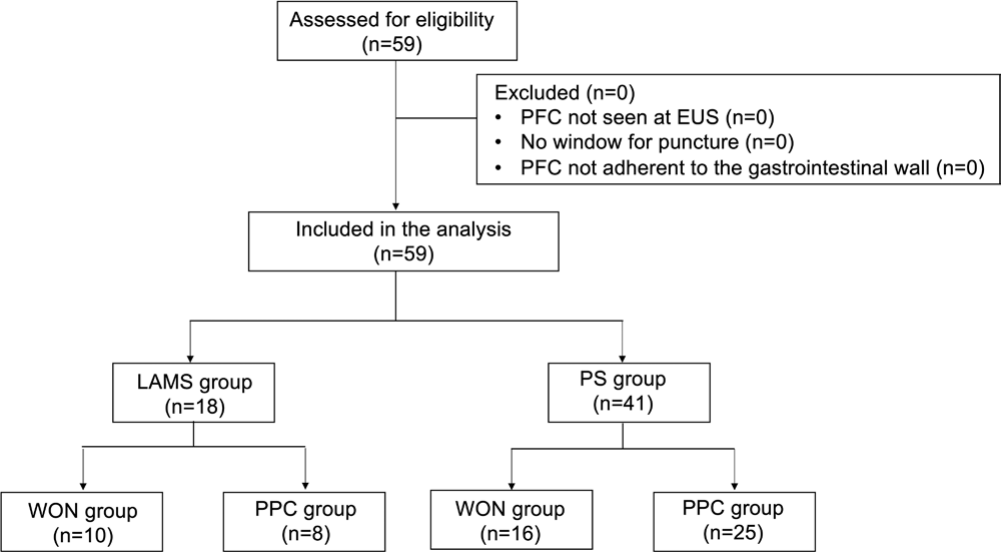
Study flowchart. LAMS = lumen-apposing metal stents, PFC = peripancreatic fluid collection, PPC = pancreatic pseudocyst, PS = plastic stent, WON = walled-off necrosis.

Figure 1 shows the patient selection and inclusion in the study. There were 18 patients in the LAMS group and 41 patients in the PS group. Patient characteristics are shown in Table 1. The maximum diameter of the PFC was significantly larger in the LAMS group (154 vs 103 mm; *P* <.001). There were no significant differences in age, sex, history of alcohol consumption or smoking, cause of PFC, presence of infection, PNI, or M-CONUT scores before the procedure.

**Table 1:** Patient characteristics and clinical details.

Variable	LAMS (n = 18)	PS (n = 41)	*P*-value
Age (yr), mean ± SD	60.1 ± 13.0	65.2 ± 10.5	.12
Gender, n (%)			
Male	13 (72.2)	31 (75.6)	.76
Female	5 (27.8)	10 (24.4)	
Smoking, n (%)	11 (61.1)	21 (51.2)	.58
Alcohol, n (%)	7 (38.9)	24 (58.5)	.26
Etiology, n (%)			
Alcohol	3 (16.7)	10 (25.0)	.74
Gallstones	1 (5.6)	3 (7.5)	>.99
Idiopathic	5 (27.8)	5 (12.5)	.26
Acute on chronic	4 (22.2)	3 (7.5)	.19
Post-surgery	1 (5.6)	10 (24.4)	.15
Others	4 (22.2)	10 (25.0)	>.99
Type of PFC, n (%)			
WON	10 (55.6)	16 (39.0)	.27
PPC	8 (44.4)	25 (61.0)	
PFC size (mm), mean ± SD	154 ± 44	103 ± 44	<.001
Infected PFC, n (%)	14 (77.8)	31 (75.6)	>.99
Pretreatment M-CONUT, mean ± SD	7.1 ± 3.3	5.8 ± 3.1	.16
Pretreatment PNI, mean ± SD	31.9 ± 8.7	34.9 ± 8.4	.22

LAMS = lumen-apposing metal stent, M-CONUT = modified controlling nutritional status, PFC = peripancreatic fluid collection, PNI = prognostic nutritional index, PPC = pancreatic pseudocyst, PS = plastic stent, WON = walled-off necrosis.

**Figure 2.**
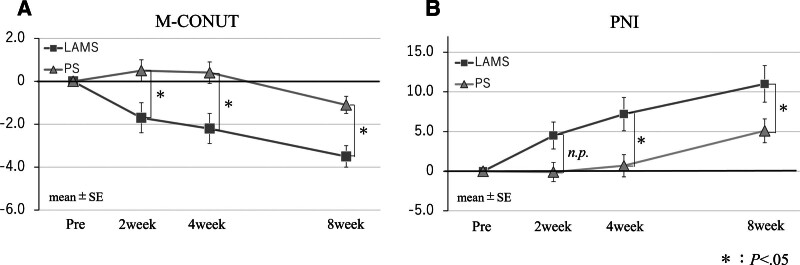
Changes in M-CONUT and PNI after treatment. (A) Changes in M-CONUT from baseline to 8 wk. (B) Changes in PNI from baseline to 8 wk. LAMS group show earlier improvement in nutritional indices (M-CONUT and PNI) than PS group. LAMS = lumen-apposing metal stents, M-CONUT = modified controlling nutritional status, PNI = prognostic nutritional index, PS = plastic stent.

The clinical outcomes are shown in Table 2. The technical success rate was 100% in both the groups. The clinical success rates were not significantly different between the 2 groups (88.9 vs 85.4%; *P* >.99). The endoscopic procedure time (21.0 vs 48.0 min; *P* <.001) and the time from initial treatment to resumption of oral intake (3.0 vs 5.0 days; *P* = .04) were significantly shorter in the LAMS group. The total number of procedures required before discharge was not significantly different between the 2 groups (2.0 vs 2.0; *P* = .68), nor was the time to discharge significantly different (28.5 vs 36.0 days; *P* = .19). The occurrence of adverse events was not significantly different between the groups (16.7 vs 14.6%; *P* >.99). Hemostasis by interventional radiology was performed in one case of bleeding. The remaining patients were treated with antibiotics, blood transfusions, and stent exchanges, and all patients were discharged. There was one death in the LAMS group and 5 deaths in the PS group during hospitalization; however, the difference was not significant (5.6 vs 12.2%; *P* = .66). The recurrence rates were similar (16.7 vs 17.1%; *P* >.99). Nutritional parameters measured using CT before and 4 weeks after treatment are shown in Table 3. Abdominal circumference, visceral fat area, subcutaneous fat area, and L3SMI were analyzed in 32 patients (11 with LAMS and 21 with PS) for whom CT imaging was available before and 4 weeks after treatment, and no significant differences were found. The post-treatment PNI showed significant improvement in the LAMS group (2 weeks: 4.5 vs −0.1; *P* = .09, 4 weeks: 7.2 vs 0.7; *P* = .04, 8 weeks: 11.0 vs 5.1; *P* = .05). Post-treatment M-CONUT scores also showed significant improvement from earlier in the LAMS group (2 weeks: −1.7 vs 0.5; *P* = .03, 4 weeks: −2.2 vs 0.4; *P* = .03, 8 weeks: −3.5 vs −1.1; *P* = .03) (Fig. 2).

**Table 2:** Clinical outcomes in the LAMS group and PS group.

Outcomes	LAMS (n = 18)	PS (n = 41)	*P*-value
Technical success, n (%)	18 (100.0)	41 (100.0)	>.99
Clinical success, n (%)	16 (88.9)	35 (85.4)	>.99
Initial procedure time (min), median (range)	21.0 (8–47)	48.0 (15–92)	<.001
Total number of procedures, median (range)	2 (1–11)	2 (1–7)	.68
Time to recommencing oral intake (d), median (range)	3 (1–11)	5 (1–29)	.04
Complications, n (%)	3 (16.7)	6 (14.6)	>.99
Bleeding	0 (0)	2 (4.9)	>.99
Stent occlusion	0 (0)	2 (4.9)	>.99
Stent migration	1 (5.6)	0 (0)	.31
Perforation of PFC	0 (0)	1 (2.4)	>.99
Infection	2 (11.1)	1 (2.4)	.64
Recurrence of PFC, n (%)	3 (16.7)	7 (17.1)	>.99
Mortality, n (%)	1 (5.6)	5 (12.2)	.66

LAMS = lumen-apposing metal stent, PFC = peripancreatic fluid collection, PS = plastic stent.

**Table 3:** Changes in nutritional parameters at 4 wk after treatment in the LAMS group and the PS group.

Parameter	LAMS (Δ, n = 18)	PS (Δ, n = 41)	*P*-value
Abdominal circumference (cm)	−4.3 ± 3.2	−2.4 ± 5.9	.38
Visceral fat area (cm^2^)	−15.1 ± 16.5	−17.7 ± 19.2	.73
Subcutaneous fat area (cm^2^)	−15.1 ± 18.9	−20.5 ± 26.2	.42
L3SMI (cm^2^/m^2^)	−4.2 ± 3.4	−4.7 ± 3.5	.61

Values are expressed as mean ± SD.

Δ **=** change from baseline, L3SMI = L3 skeletal muscle index, LAMS = lumen-apposing metal stent, PS = plastic stent.

Subgroup analyses according to the type of PFC are shown in Table S1, Supplemental Digital Content, https://links.lww.com/MD/R511.

No significant interaction was found, although in the PPC subgroup LAMS was associated with earlier oral intake and greater improvement in nutritional indices compared with PS.

## 4. Discussion

Currently, EUS-TD is widely used as a minimally invasive treatment for PFC. However, a standard technique, including the choice of stent type, has not yet been established. The LAMS was developed as a dedicated device for EUS-TD, and its usefulness and safety were reported by Itoi et al in 2012.^[11]^ Since the development of LAMS, many comparisons of treatment outcomes between conventional PS and FCSEMS have been conducted.

Clinical success rates have been examined in several randomized controlled trials and meta-analyses; however, no consensus has been reached. This may be because the definitions of clinical success and the EUS-TD procedure are not standardized among hospitals. Regarding adverse events, LAMS had a high frequency of bleeding complications when it was first developed. However, measures such as early stent removal and the use of a double-pigtail-type PS have been adopted, and a recent meta-analysis reported no differences in bleeding complications between LAMS and PS.^[24]^ In our study, there were no differences in the clinical success rates and adverse events between the LAMS and PS groups. However, it is possible that the LAMS group had larger cyst diameters, which may have affected the outcomes.

In this study, we compared the variations in nutritional status after EUS-TD according to the type of stent used. The PNI and CONUT score are simple and objective nutritional indices calculated from blood tests. Various methods for measuring the PNI have been proposed; however, the PNI by Onodera et al, which is calculated from serum albumin levels and lymphocyte counts, is often used in Japan. Onodera et al developed the PNI in 1984 as a predictive index for the risk of postoperative complications; however, in recent years, it has also been used as a general nutritional status evaluation index owing to its simplicity.^[18]^ The CONUT score is a nutritional index calculated from serum albumin levels, total cholesterol levels, and TLCs.^[19]^ In addition to screening for nutritional status, the CONUT score is useful in predicting the prognosis of patients with infectious diseases, inflammatory diseases, cardiovascular diseases, malignant tumors, and after surgery.^[25–28]^ In Japan, Takahashi et al proposed the M-CONUT score, in which the total cholesterol level was replaced with the hemoglobin concentration, and a good correlation with the CONUT score has been demonstrated.^[20]^ In this study, we selected the M-CONUT score, which has fewer missing values, to evaluate the changes in nutritional indices over time. In our study, both the LAMS and PS groups were malnourished in terms of the PNI and CONUT score before the procedure. The LAMS group was able to resume enteral feeding earlier after the procedure than the PS group. In addition, the PNI and M-CONUT score showed greater improvements in the LAMS group. These results suggest that LAMS contributes to the early improvement of nutritional status in patients with PFC. This may be due to the higher drainage efficiency of the LAMS, which results in more rapid resolution of inflammation and reduction of PFC. In contrast, no significant differences in body fat or skeletal muscle mass between the 2 groups at 4 weeks after treatment, which may be due to the limited number of patients and the short observation period. Exploratory subgroup analyses suggested that potential benefits of LAMS over PS were more apparent in patients with PPC, while no clear differences were observed in those with walled-off necrosis. These results should be interpreted cautiously due to limited number of patients. No studies have reported changes in nutritional status after EUS-TD, and the finding that LAMS contributes not only to early improvement in the pathophysiology of the PFC, but also to early improvement in nutritional status is new.

Early nutritional improvement after treatment for PFC may lead to improved long-term prognosis, including the prevention of infection relapse. This study is the first to suggest the usefulness of LAMS from a nutritional perspective. However, it did not show a significant effect in preventing recurrent infections. The fact that this study was a retrospective analysis, the number of stents, whether PS was implanted in the LAMS, and the duration of LAMS implantation were not standardized among hospitals, and that the observation period was short may have contributed to the lack of efficacy in preventing recurrent infections. To overcome these issues, it is necessary to standardize the procedure and conduct a prospective RCT involving a large number of patients, which may accurately evaluate whether stent selection during EUS-TD leads to not only improved nutritional status after the procedure but also improved long-term prognosis, such as the prevention of PFC infection flare-ups. This issue should be addressed in future studies.

This study has several limitations. First, it was a retrospective observational study, which may have introduced selection bias and unmeasured confounding factors. Second, the sample size was relatively small, particularly in the subgroup analyses, which may have reduced the statistical power to detect meaningful differences. Third, procedural factors, including the number of stents used, concomitant placement of PS in the LAMS group, and duration of stent indwelling, were not standardized across participating centers, which could have influenced treatment outcomes. Fourth, the follow-up period was relatively short, and therefore, long-term effects on nutritional status and infection recurrence could not be adequately assessed. Consequently, the results should be interpreted with caution, and further large-scale prospective studies with standardized protocols are warranted.

## 5. Conclusion

In this study, the LAMS group showed earlier improvement of nutritional status than the PS group. LAMS may contribute to early recovery in patients with PFC, although future prospective studies are needed to confirm its long-term clinical impact.

## Acknowledgments

This study was conducted with the cooperation of the Yamaguchi Clinical Research Network for Pancreatobiliary (YCR-P), and we sincerely acknowledge their contribution.

## Author contributions

**Conceptualization:** Takanori Tsuyama, Shigeyuki Suenaga.

**Data curation:** Takanori Tsuyama.

**Investigation:** Takanori Tsuyama, Shigeyuki Suenaga, Harumi Suehiro, Ukyo Shinagawa, Kaori Hamamoto, Shoko Tabara, Shuhei Shinoda, Shogo Amano, Manabu Sen-Yo, Noriko Ishigaki, Michitaka Kawano, Hirofumi Harima.

**Project administration:** Shigeyuki Suenaga.

**Supervision:** Taro Takami.

**Writing – original draft:** Takanori Tsuyama.

**Writing – review & editing:** Shigeyuki Suenaga.

## Supplementary Material
