# Supplementary material for: Lumen-apposing metal stent for peripancreatic fluid collection contributes to early improvement of nutritional status: A multicenter retrospective study (YCR-P001)
Source: Medicine (Baltimore). 2026 Mar 13;105(11):e48020. doi: 10.1097/MD.0000000000048020 (PMC12991500; doi:10.1097/MD.0000000000048020)
Supplement: Supplementary file 1 [file medi-105-e48020-s001.docx]

**Table S1 – Subgroup analysis of clinical outcomes by type of peripancreatic fluid collection.**

| **Outcome** | **PPC subgroup** | | | **WON subgroup** | | | ***P* for interaction** |
| --- | --- | --- | --- | --- | --- | --- | --- |
|  | **LAMS**  **(n=8)** | **PS**  **(n=25)** | ***P* value** | **LAMS**  **(n=10)** | **PS**  **(n=16)** | ***P* value** |  |
| Clinical success, n (%) | 8 (100.0) | 23 (92.0) | >.99 | 8 (80.0) | 12 (75.0) | 1.000 | .38 |
| Time to recommencing oral intake (days), median (range) | 1.5 (1-9) | 4.5 (1-20) | .04 | 4.0 (1-11) | 9.0 (1-59) | 0.125 | .28 |
| M-CONUT change, mean±SD | -3.7±2.7 | 0.5±3.4 | .01 | -1.0±3.6 | 0.3±3.5 | 0.495 | .16 |
| PNI change, mean±SD | 11.5±7.1 | 0.6±9.2 | .01 | 4.1±11.8 | 0.4±7.4 | 0.673 | .18 |
| PPC = pancreatic pseudocyst, WON = walled-off necrosis, LAMS = lumen-apposing metal stent, PS = plastic stent, PFC = peripancreatic fluid collection, M-CONUT = modified controlling nutritional status, PNI = prognostic nutritional index. | | | | | | | |
